# Molecular Epidemiology of the HIV-1 Subtype B Sub-Epidemic in Bulgaria

**DOI:** 10.3390/v12040441

**Published:** 2020-04-14

**Authors:** Ivailo Alexiev, Ellsworth M. Campbell, Sergey Knyazev, Yi Pan, Lyubomira Grigorova, Reneta Dimitrova, Aleksandra Partsuneva, Anna Gancheva, Asya Kostadinova, Carole Seguin-Devaux, William M. Switzer

**Affiliations:** 1 National Reference Laboratory of HIV, National Center of Infectious and Parasitic Diseases, Sofia 1233, Bulgaria; lyubomiragrigorova@gmail.com (L.G.); naydenova.reneta@gmail.com (R.D.); alexandra.partsuneva@gmail.com (A.P.); gancheva.anna@gmail.com (A.G.); deshova.asi@gmail.com (A.K.); 2 Division of HIV/AIDS Prevention, National Center for HIV/AIDS, Viral Hepatitis, STD, and TB Prevention, Centers for Disease Control and Prevention, Atlanta, GA 30303, USA; ykk7@cdc.gov (E.M.C.); nvr5@cdc.gov (S.K.); jnu5@cdc.gov (Y.P.); bis3@cdc.gov (W.M.S.); 3 Department of Computer Science, Georgia State University, Atlanta, GA 30303, USA; 4 Oak Ridge Institute for Science and Education, Oak Ridge, TN 37830, USA; 5 Department of Infection and Immunity, Luxembourg Institute of Health, Esch-sur-Alzette, L-4354 Esch-sur-Alzette Luxembourg; carole.devaux@lih.lu

**Keywords:** HIV-1, molecular epidemiology, transmission clusters, subtype, drug resistance, prevention

## Abstract

HIV-1 subtype B is the predominant strain in Bulgaria, yet little is known about the molecular epidemiology of these infections, including its origin and transmissibility. We used a phylodynamics approach by combining and analyzing 663 HIV-1 polymerase (*pol*) sequences collected from persons diagnosed with HIV/AIDS between 1988–2018 and associated epidemiologic data to better understand this sub-epidemic in Bulgaria. Using network analyses at a 1.5% genetic distance threshold (*d)* we found several large phylogenetic clusters composed mostly of men who have sex with men (MSM) and male heterosexuals (HET). However, at d = 0.5%, used to identify more recent transmission, the largest clusters dissociated to become smaller in size. The majority of female HET and persons with other transmission risks were singletons or pairs in the network. Phylogenetic analysis of the Bulgarian *pol* sequences with publicly available global sequences showed that subtype B was likely introduced into Bulgaria from multiple countries, including Israel and several European countries. Our findings indicate that subtype B was introduced into Bulgaria multiple times since 1988 and then infections rapidly spread among MSM and non-disclosed MSM. These high-risk behaviors continue to spread subtype B infection in Bulgaria as evidenced by the large clusters at *d* = 0.5%. Relatively low levels of antiretroviral drug resistance were observed in our study. Prevention strategies should continue to include increased testing and linkage to care and treatment, as well as expanded outreach to the MSM communities.

## 1. Introduction

HIV-1 consists of four major phylogenetic groups: M (major), N (new), O (outlier), and P, representing independent cross-species transition from SIV in chimpanzee and gorillas to HIV in humans [1]. HIV-1 group M is responsible for the current pandemic and comprises genetically distinct subtypes (A, B, C, D, F, G, H, J, K, and, more recently, L), 101 circulating recombinant forms (CRFs) to date, and numerous unique recombinant forms (URFs) [2,3].

HIV-1 subtypes are unequally distributed globally, which has been explained by different founder effects followed by local spread driven by socioeconomic and behavioral factors and circulation within and between specific risk groups [4,5,6]. Subtype C is the most abundant strain worldwide and is prevalent in South and Eastern Africa and South East Asia [2]. Subtype B is predominant in North America, Western Europe, and Australia, while subtype A predominates in Eastern Europe and central Asia, including Russia [2,7]. CRFs and URFs are widely distributed in central Africa and in countries where different subtypes co-circulate [2,8,9]. The wide variety of subtypes, which are distributed mainly in different populations, may be used for molecular epidemiological analysis to track and understand the dynamics and patterns of HIV-1 transmission and for developing strategic prevention programs [10].

The HIV-1 epidemic in Bulgaria has been affected by various historic and socio-economic factors. Multiple HIV-1 subtypes and recombinant forms have been introduced from various countries of the world and subsequently disseminated unequally among individuals from different transmission groups, including heterosexual (HET) persons, men who have sex with men (MSM), and persons who inject drugs (PWID) [11,12,13,14,15,16]. Due to random founder events, certain HIV-1 strains have spread rapidly after being introduced into high risk groups and then expanded into transmission clusters, representing local outbreaks. Indeed, such events were observed since 2005 when two different CRFs (CRF01_AE and CRF02_AG) have been independently introduced and rapidly disseminated among two geographically distinct subgroups of PWID, resulting in local outbreaks [12,16].

Although various subtypes were initially introduced in the country, subtype B is the most widespread and is found in almost half of HIV-1-infected persons in Bulgaria. Furthermore, subtype B is more prevalent among MSM and represents a significant proportion of the current HIV epidemic in Bulgaria. Since 2005, there has been a sharp increase in HIV incidence among MSM leading to an outbreak with significant involvement of subtype B viruses in the context of diverse subtypes and CRFs in other risk groups in Bulgaria, including PWID. The purpose of the current study was to characterize the underlying HIV transmission dynamics of the subtype B epidemic in Bulgaria by integrating and analyzing available HIV-1 epidemiologic and nucleotide sequence data by using a phylodynamics approach to help inform prevention efforts.

## 2. Materials and Methods

### 2.1. Study Design and Specimen Preparation

Fresh, whole blood samples from persons diagnosed with HIV-1 between 1988 and 2018 were collected during the diagnostic process and clinical follow-up at the National Reference Confirmatory Laboratory of HIV (NRCL of HIV) Sofia, Bulgaria. Patient self-assessment interviews were conducted during the diagnostic process to obtain demographic and clinical information in accordance with national regulations. Plasma samples were linked to demographic and clinical data through an anonymous numerical code in accordance with the ethical standards of Bulgaria as previously described [14]. This study was approved by the Ethical Committee at the National Centre of Infectious and Parasitic Diseases, Sofia, Bulgaria (NCIPD IRB 00006384, 03.February.2020). A non-research determination (protocol # 6832) was approved at CDC for analysis of the HIV sequences in this study. Viral RNA was extracted from plasma samples using the Abbott Viroseq HIV-1 Genotyping Test.

### 2.2. Data Set and Sequence Analyses

The HIV-1 protease and reverse transcriptase regions of the *pol* gene was sequenced using the ViroSeq HIV-1 Genotyping Test (Abbott, Chicago, IL, USA) and/or TruGene DNA Sequencing System (Siemens Medical Solutions Diagnostics, Germany) and either the Applied Biosystems 3130xl genetic analyzer or an OpenGene DNA sequencing system following the manufacturer’s protocol [16].

HIV-1 subtype was determined using the automated subtype identification tool COMET v2.2 [17], the REGA HIV-1 subtyping tool version 3.0 [18] and the jumping profile Hidden Markov Model (jpHMM) [19]. Only sequences that were identified as pure subtype B viruses were included in the current study. HIV-1 drug resistance mutations (DRMs) were determined according to the WHO 2009 SDRM list [20] using the Genotypic Resistance Interpretation Algorithm of Sierra v2.4.2 of the Stanford University HIV Drug Resistance Database (https://hivdb.stanford.edu/hivdb/by-sequences/) [21].

Sequence alignments were performed using the MUSCLE algorithm implemented in AliView version 1.23 [22,23]. Additional quality control of the subtype purity and possible presence of sequence gaps was performed. After the manual editing and preliminary quality analysis, the complete dataset contained 663 HIV-1 subtype B Bulgarian sequences. All Bulgarian HIV-1 strains were deposited in GenBank (Supporting Document 1).

Identification of subtype B clusters and characterization of the transmission network was done using the sequence alignment and MicrobeTrace (http://github.com/cdcgov/microbetrace) [24] at Tamura–Nei genetic distance (*d*) cutoffs of 0.005 (0.5%) and 0.015 (1.5%) nucleotide substitutions/site. Graphically, transmission linkages are represented by lines drawn between both nodes, where each node represents a participants’ *pol* sequence. If a participants’ *pol* sequence was linked to another according to a specific threshold, both participants were labeled as clustered. Those participants whose *pol* sequence did not link to any other participant were labeled unclustered. Categorical and numeric assortativity coefficients for selected variables were calculated using the Python package https://github.com/Sergey-Knyazev/attribute_assortativity [25] NetworkX (https://networkx.github.io/) [26] and thresholds of d ≤ 0.5% and 1.5%.

Identification of the potential origin of subtype B viruses in Bulgaria was evaluated by phylogenetic analyses. Approximate maximum likelihood (ML) phylogenies were constructed using all 663 Bulgarian *pol* sequences, and the top BLAST hits at GenBank to the Bulgarian sequences (*n* = 248) and HIV-1 *pol* sequences in the Los Alamos database from 2018 (*n* = 1684), excluding any duplicates, using the GTR nucleotide substitution model in FastTree v2.1.10 [27].

### 2.3. Statistical Analysis

Epidemiological characteristics, such as gender, age, country of origin, likely country of infection, region in Bulgaria, and transmission categories, were considered. The frequencies as well as percentages were analyzed by subtype B infection and non-subtype B infection groups. The association between subtype B infection and the characteristics were evaluated by the chi-squared test or Fisher’s exact test when sample sizes were small.

## 3. Results

### 3.1. Characteristics of the Subtype B Infections in Bulgaria

**Figure 1:**
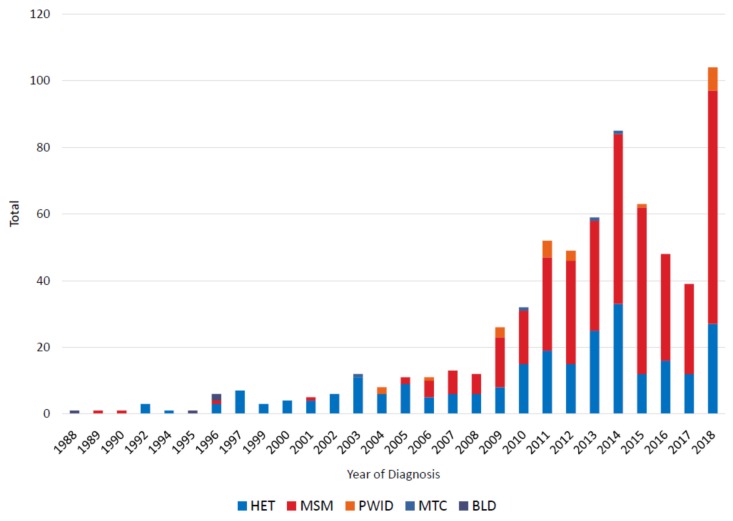
Noncumulative HIV-1 subtype B diagnoses in Bulgaria from 1988 to 2018 by transmission category. HET, heterosexual; MSM, men who have sex with men; PWID, persons who inject drugs; MTC, mother-to-child; BLD, blood transfusion.

A total of 663 HIV-1 subtype B infections were identified from 1988 to 2018 (Table 1). The first subtype B infections in Bulgaria were diagnosed in 1988, then steadily increased until 2014 when 85 cases were identified, followed by a decline until 2018 when 104 new cases were identified (Figure 1). The initial cases in the epidemic were mostly in persons receiving blood transfusions (BLD) or via HET transmission. Between 1989 and 2004 the number of HIV diagnoses was rare (one or none per year) in MSM and then increased rapidly thereafter to 70 new cases in 2018 for a total of 377 MSM (includes one person reporting MSM and PWID). There were 256 total HET infections. HIV-1 subtype B diagnoses in PWID (*n* = 22), from mother-to-child (MTC, *n* = 4), and transmission by contaminated blood (*n* = 4) were rare during this study period. There were 593 males and 70 females with subtype B infection. Age at diagnosis ranged from 1 to 73 and, based on patient interviews 585 infections presumed to have occurred in Bulgaria whereas 78 occurred in other countries, mostly Europe (*n* = 63).

Among subtype B infected participants, 593 out of 663 (89.4%) were males while among the non-subtype B infected participants, 612 out of 819 (74.7%) were males. The percentage for male participants with subtype B infection was statistically higher than those with non-subtype B infection (*p* < 0.0001, Table 1, chi-squared test). The percentage of participants with subtype B infection who were likely infected in Bulgaria (88.2%) was like that among participants with non-subtype B infection (86%, *p* = 0.195, Table 1, chi-squared test). We found that participants with subtype B infection had lower percentages of heterosexual (38.6% vs 44.6%) transmissions compared to non-subtype B participants, higher percentages of MSM (56.7% vs. 20.3%) compared to non-subtype B participants, and much lower percentages of PWIDS (3.3% vs. 30.9%) compared to non-subtype B participants (Table 1). The overall distributions of transmission risks between subtype B and non-subtype B were different (*p* < 0.0001, Table 1, Fisher’s exact test).

### 3.2. Identification and Characterization of HIV-1 Subtype B Transmission Clusters in Bulgaria

**Figure 2:**
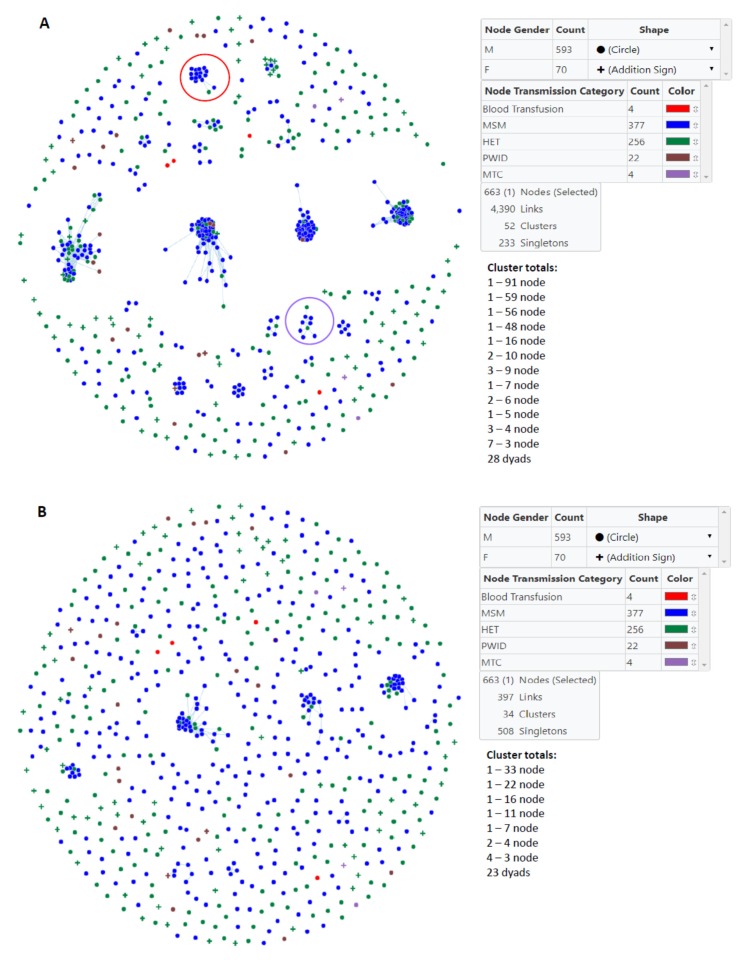
Inference of subtype B clusters in Bulgaria using MicrobeTrace. (**A**) A total of 52 clusters were identified using a genetic distance of 1.5% compared to (**B)** 34 clusters at a genetic distance of 0.5%. Gender is indicated by circles (male) and addition signs (female). Transmission category is indicated with color (red, blood transfusion; blue, men who have sex with men (MSM); green, heterosexual (HET); brown, persons who inject drugs (PWID); purple, mother-to-child (MTC). One person reporting MSM and PWID risks was included in the MSM group. Cluster totals by node (members) and total number of links in the transmission network is provided. Large clusters with most of the members have HIV infection with drug resistance mutations at the 1.5% threshold are circled in red (nonnucleoside reverse transcriptase inhibitors) and purple (protease inhibitors).

We used MicrobeTrace to infer HIV transmission clusters at two genetic distance thresholds to capture genetically related established and recent infections (1.5%) and then to identify more recent transmission linkage (0.5%) within Bulgaria (Figure 2). At the 1.5% cutoff, 52 clusters were inferred, including 24 clusters of sizes 3 or more and 28 dyads (Table 2, Figure 2A). Overall 68.8% of the *pol* sequences from men and 31.4% of those from women fell into transmission clusters. Only one cluster with 10 members had more females than males (60% vs. 40%, respectively). The largest clusters at the 1.5% threshold with >3 members were composed of mostly MSM (20–100%) (Table 2, Figure 2A). Although the three largest clusters with sizes 56, 59, and 91 members each had the highest numbers of HETs and PWIDs, several of the smaller clusters had higher percentages of HET and PWID, and four small clusters consisted of only MSM (Table 2, Figure 2A). Within the three large clusters there were only small numbers of women (*n* = 4, 0, or 1, respectively). Among clusters ≤3 members in size and singletons (unclustered persons), the highest percentages of MSM, HET, and PWID were in triads, singletons, and dyads, respectively (Table 2). Most women in our study (*n* = 48) were not linked to any other cases (Table 2).

At the more stringent 0.5% cutoff only 34 clusters were inferred of which 11 were ≥3 members in size and 23 were dyads (Table 3, Figure 2B). The large 91, 59, 56, and 48 member clusters at the 1.5% genetic distance cutoff were reduced to 33, 11, 22, and 16 member clusters at the 0.5% threshold, respectively. Similarly, three smaller clusters of sizes 9, 7, and 5 became clusters of sizes 7, 4, and 4, respectively.

### 3.3. Assortative Mixing of Pairs, Gender, Similar Ages, Transmission Category, and Geographic Location in the Subtype B Transmission Networks

Assortativity is a quantitative measure, often used to help characterize cluster composition, that describes the likelihood that a node in a network is connected to a node bearing similar characteristics. Assortativity coefficient (*r*) values of 1.0 indicate perfect assortativity, while at *r* = −1.0 the network is completely disassortative, and at *r* = 0 the network is non-assortative. Our analysis found that dyads at both *d* = 0.5% and 1.5% were strongly assortative for sampling region within Bulgaria (0.3442 and 0.6452, respectively) and transmission category (0.4889 and 0.6474, respectively) (Table 4). However, as cluster size increases beyond dyadic pairs, sampling region within Bulgaria and the transmission category were non-assortative. Gender was assortative at *d* = 0.5% for pairs, and clusters ≥10 in size at *d* = 1.5% and for all clusters at both genetic distances (Table 3). Age was assortative for d = 1.5% for pairs and clusters with 3–9 members.

### 3.4. Origin of Subtype B Infections in Bulgaria

**Figure 3:**
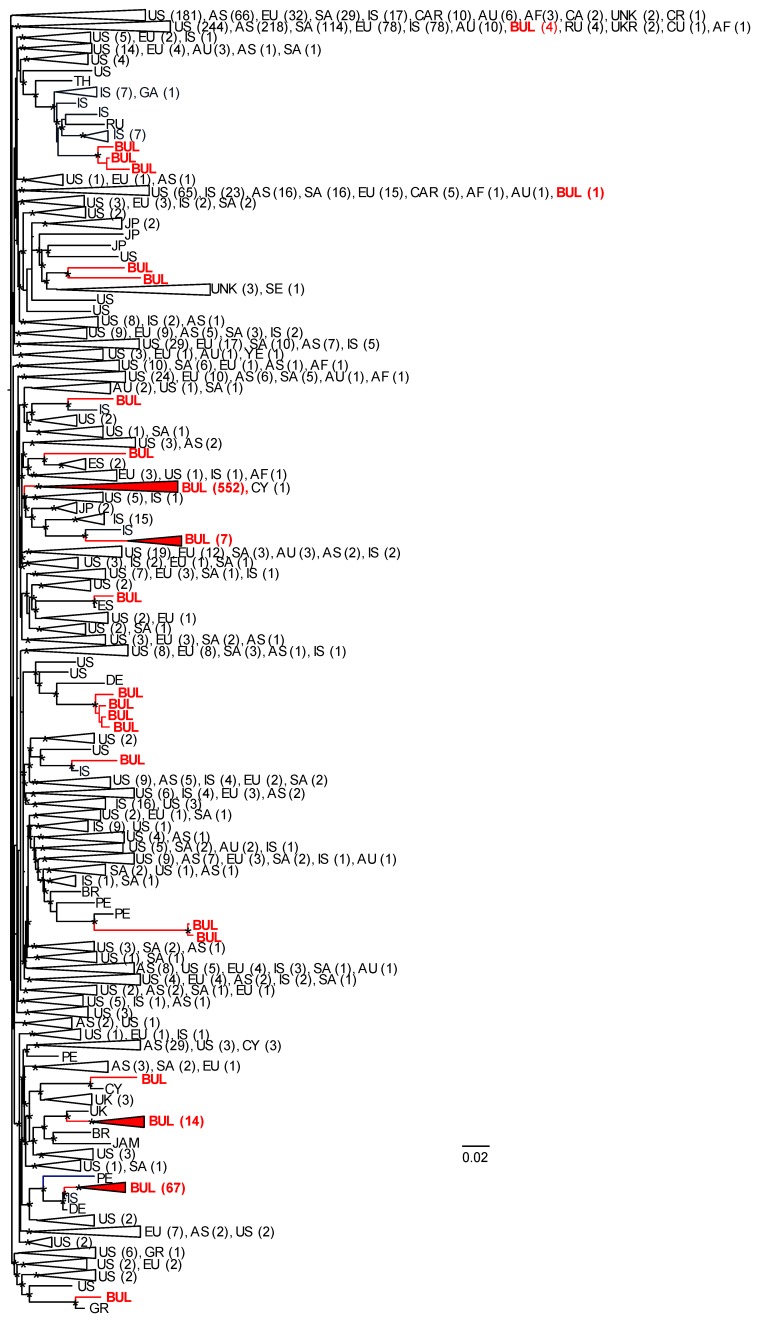
Maximum-likelihood (ML) phylogeny of global HIV-1 subtype B sequences. The ML tree was constructed with FastTree v2.1.10 using 663 sequences from Bulgaria and 1933 global sequences. Confidence values of clusters were assessed by using the Shimodaira–Hasegawa (SH) test in FastTree. SH values > 0.7 are shown at nodes with an asterisk. Large clades are collapsed, and the corresponding country or continent are provided with codes followed by total numbers of sequences in parentheses. AF, Africa; AS, Asia; AU, Australia; BR, Brazil; BUL, Bulgaria (in red text); CA, Canada; CAR, Caribbean; CR, Costa Rica; CU, Cuba; CY, Cyprus; DE, Germany; ES, Spain; EU Europe; GA, Georgia; GR, Greece; IS, Israel; JAM, Jamaica; JP, Japan; PE, Peru; SA, South America; SE, Sweden; RU, Russia; TH, Thailand; UKR, Ukraine; UK, United Kingdom; UNK, unknown; US, United States; YE, Yemen.

A total of 2596 global HIV-1 *pol* sequences were used to infer the phylogenetic relationships of Bulgarian subtype B sequences with those from other countries to evaluate potential origins of the subtype B sub-epidemic in Bulgaria (Figure 3 and the Newick file is provided in Supplementary Materials). Sequences immediately ancestral to those from Bulgaria with strong support (Shimodaira–Hasegawa (SH) values ≥ 0.7) were considered potential sources of infections to Bulgaria with the common caveat of molecular epidemiology investigations that not all infected persons are sampled. A total of 20 different phylogenetic clusters containing Bulgarian sequences were inferred ranging from pairs (*n* = 6) and triads (*n* = 5) to a large clade of 552 Bulgarian sequences, as well as one sequence from Cyprus. The majority of clades containing at least two Bulgarian sequences (6/8, 75.0%) consisted of sequences from outside Bulgaria that were always ancestral to the Bulgarian sequences and originated from multiple countries, including Israel, Germany, Great Britain, Russia, Japan, Brazil, Peru, and the U.S. For example, immediately ancestral to the strongly supported (SH = 0.98) largest Bulgarian clade (*n* = 552) was a clade (SH = 0.75) consisting of 31 total sequences, including those from the U.S. (*n* = 5), Japan (*n* = 2), Israel (*n* = 17), and Bulgaria (*n* = 7). Within this 31 taxa clade all non-Bulgarian sequences were ancestral to the seven Bulgaria sequences with all 17 sequences from Israel as the closest sister clade (SH = 0.91). Immediately ancestral to the second largest clade of Bulgarian sequences (*n* = 67, SH = 0.99) was a single sequence from Israel from 2010 (SH = 0.81), followed by a single sequence from Germany from 2010 (SH = 0.98), a single sequence from Peru from 2014 (SH = 0.9), and two sequences from the U.S. (SH = 0.8). Immediately ancestral to the next largest Bulgarian clade (*n* = 14, SH = 0.98) was a single sequence from Great Britain from 2014 followed by a sequence from Brazil from 2010 and one from the U.S. from 2009 (SH = 0.81). Four Bulgarian sequences shared ancestry with one sequence from Germany (SH = 0.91), while another three Bulgarian sequences clustered with 16 taxa from Israel, one from Russia, and one from Germany (SH = 0.98). In the other two clusters with two Bulgarian sequences, the Bulgarian sequences were internal to non-Bulgarian sequences from Japan, Senegal, the U.S. Peru, Brazil, or the origin was not provided. Only one clade of three taxa had the single Bulgarian *pol* sequence ancestral to non-Bulgarian sequences, which were both from Spain. 

A total of 78 (11,8%) persons reported acquiring their infections outside of Bulgaria. Of these 78 infections, the majority (63/78, 80.8%) clustered within the large 553 taxa Bulgarian + Cyprus clade, of which only two were ancestral to subclades of 96 and 16 members each. Sequences from five persons within the 67 taxa clade (7.5%) reported acquiring their infections abroad, three within each of the 7 (42.9%) and 14 (21.4%) taxa clades. Two were within three member clades containing sequences from Brazil or Spain, one was paired with a sequence from Cyprus, and one shared a common ancestor with nine other sequences from Bulgaria (*n* = 1), Japan (*n* = 4), Senegal (*n* = 1), the U.S. (*n* = 1), or the country origin was not provided (*n* = 3). There were equal numbers of HET (*n* = 36, 10 females, 26 male) and MSM (*n* = 39) among these 78 persons, with only three male PWIDs.

### 3.5. Low Prevalence of Drug Resistance Mutations in Transmission Clusters

We identified 70 resistance mutations in the study population, including 13 to protease inhibitors (PIs), 33 to nucleoside reverse transcriptase inhibitors (NRTIs), and 23 to non-nucleoside reverse transcriptase inhibitors (NNRTIs) (Table S1). The most prevalent PI, NRTI, and NNRTI mutations were L90M (21/44, 47.7%), M184V (17/95, 17.9%), and E138A (26/100, 26.0%), respectively. A total of 28 patients had resistance mutations to PIs; of these, two individuals had four mutations, two had three, six had two, and 18 had single PI mutations. A total of 41 patients had mutations to NRTIs; of these, one patient had nine mutations, one had seven mutations, four had six mutations, one had five mutations, three had four mutations, one had three, five had two, and 25 patients had one mutation each. A total of 97 individuals had mutations to NNRTIs; of these, one patient had three mutations, 12 had two mutations and 84 had one mutation each. A total of 24 patients had resistance mutations to more than one class of antiretroviral drugs, 16 individuals to PIs + NRTIs, seven to PIs + NNRTIs, 11 to NRTIs + NNTRIs, and five to all three classes PIs + NRTI + NNRTIs. 

Overall, at *d* = 1.5% there were more DRMs in sequences classified as singletons (95/233, 40.8%) and dyads (17/56, 30.4%) compared to clusters ≥3 members in size (54/374, 14.4%); the majority being NNRTI DRMs (97/663, 14.6%) (Table 2). For two clusters with 16 and nine members the majority had DRMS (Table 2, Figure 2A). In the 16-member cluster (15 MSM, 1 HET), 10 MSM (62.5%) had E138G, one MSM (6.3%) with the V179E, two MSM (12.5%) with both E138G and V179E, and one MSM (6.3%) with the K101E and E138G NNRTI DRMs. One MSM and one HET in this 16-member cluster did not have any major DRMs. In one 9-member cluster (7 MSM, 2 HET) all persons had the L90M PI DRM; two (1 MSM,1 HET) also had the V106I NNRTI DRM. However, at *d* = 0.5%, the 16-member cluster with NNRTI DRMs dissociated into singletons and two MSM pairs; one pair with the E13G8/V179E DRMs and the other with the E138G only DRM. Likewise, at the 0.5% threshold the 9-member cluster with the L90M PI DRM dissociated into singletons and one MSM pair. In linked pairs at the 1.5% genetic distance, the major NNRTI DRMs were V179D/E (5/13, 38.5%) and E138A/E/G (8/13, 61.5%), and these were found in four MSM, six HET, and two PWID. Fifteen persons whose HIV did not cluster had PI DRMs of which the majority were M46I and/or L90M. Fourteen of these 15 persons (93.3%) also had NRTI DRMs and three also had NNRTI DRMs. Sixteen additional singletons had NRTI DRMS, of which three also had NNRTI DRMs. Twenty-three unclustered persons had only NRTIs, most of which are E138A/E (12/23, 52.1%), K103N/S (4/23, 17.4%), or V106I (3/23, 13.0%).

## 4. Discussion

Identifying at-risk individuals and behaviors through epidemiological contact tracing has been a successful strategy in controlling many infectious diseases [10]. More recently, combining traditional epidemiological data with pathogen sequences and conducting phylogenetic and/or network analysis has provided a more holistic understanding of pathogen transmission networks, including HIV [28]. Furthermore, studies have shown that inclusion of HIV sequence data also uncovers hidden linkages within the transmission network not identified with contact tracing alone [29]. We used phylogenetic and network methods to investigate transmission networks of HIV-1 subtype B, the most prevalent HIV strain in Bulgaria, by integrating all available HIV-1 subtype B sequences through 2018 with the associated demographic and epidemiologic data.

Although the first case of subtype B infection in Bulgaria was diagnosed in a person who received a blood transfusion, subsequent diagnoses were mostly heterosexual transmission cases until 2006 when more diagnoses were found in MSM [14]. Subtype B infections have since increased significantly in MSM and HET with much lower prevalence in PWID and children born to infected mothers [16]. In 2018, 53.1% of new diagnoses were MSM compared to 34.7% HET, 11.3% PWID, and 1% vertical transmissions. The predominance of subtype B infections in MSM in Bulgaria now resembles epidemics in many Western European countries and North America [30]. These findings contrast with the non-B infections in Bulgaria, which is composed mostly of PWIDs and heterosexual transmissions. Indeed, we previously reported that local, independent HIV-1 outbreaks were observed in PWID in two separate geographical regions of the country by two different recombinant forms of HIV-1, CRF01_AE, and CRF02_AG [16]. These CRF outbreaks in PWIDS resulted in major public health responses by the Bulgarian Ministry of Health, including HIV prevention and education campaigns, which significantly reduced HIV diagnoses in this vulnerable population. However, despite significant efforts by public health programs and NGOs to provide educational and prevention campaigns, HIV continues to be a significant burden for MSM in Bulgaria and other European countries [31,32,33]. 

Network analysis at a 1.5% genetic distance identified 24 clusters with at least three members of which the majority consisted of MSM or male HET. These results confirm the spread of subtype B infections within MSM but also show that men identifying as HET may also be sharing MSM behaviors (i.e., non-disclosed MSM). Although we did not capture information about bisexuality, our finding that most HIV-1-infected women were singletons support this hypothesis. Only one 10-member cluster contained predominantly females of which eight were HET (six females, two male) and two were MSM, more likely indicative of a cluster composition containing persons with bisexual practices. Similar situations have been reported elsewhere in Europe and the US where subtype B predominates [34,35]. Prevention efforts to reduce stigma may help to improve reporting of potential transmission risks and increase acceptability of treatment and care, including PrEP [36,37]. There were nearly equal numbers of MSM and HET members in the clusters composed of only two or three sequences at the 1.5% threshold. More HET, PWID, blood transfusion recipients, and vertical transmissions did not cluster, indicating these were dead-end transmission events because the source of the infection died, was not identified, or an HIV-1 *pol* sequence was not obtained. Another possibility is that the infection was acquired abroad, which has been reported by 11.8% of the patients in our study. Look-back studies for persons infected by blood transfusions further limited spread of these infections during the first years of the epidemic in Bulgaria [14].

Due to the relatively constant mutation rate and evolution of HIV, the different genetic distance thresholds can inform the timing of the probable infection and thus determine whether patients are infected with a genetically similar or distant virus [38]. Lowering the genetic distance threshold to 0.5% in the network analysis to identify more recent transmissions reduced the number of clusters with >3 members by 58.8% and decreased the number of triads and dyads by 42.9% and 17.9%, respectively. For example, the largest cluster of 91 members at the 1.5% threshold only contained 33 persons at the 0.5%. In addition, the lower threshold also further reduced the number of women in the clusters though 0–27% of the men self-identified as HET in these clusters. At *d* = 0.5% and 1.5%, *pol* sequences from MSM were more likely to participate in transmission clusters while sequences from HET and PWID were less frequently included in clusters. Moreover, clusters formed predominantly by MSM contained more members than clusters dominated by HET. Combined, these results indicate that most subtype B infections are likely in MSM with established infections, which consequently can facilitate further expansion of these large clusters by the addition of more recent MSM transmissions. Indeed, we have identified likely growth of the established MSM clusters with recent infections at the 0.5% genetic distance. Our findings likely reflect that the increased outreach and testing campaigns in Bulgaria have identified previously undiagnosed HIV-1 cases that contribute to the larger cluster sizes at the 1.5% genetic distance. Nonetheless, since the large clusters at the 0.5% threshold represent recent and rapidly growing transmission clusters among MSM and possibly bisexual men, additional prevention strategies targeting MSM and bisexual men will be beneficial.

The assortativity analyses illustrated quantitative differences between dyads (size = 2) and larger clusters (size = 3–9 or size ≥ 10) with respect to the sampling region and transmission category, at stringent (d ≤ 0.5%) and relaxed (d ≤ 1.5%) genetic distance thresholds. Dyads were overwhelmingly likely to represent transmission events within the same geographic region and within the same transmission category. In contrast, as clusters grew to include three or more members, cluster composition varied with respect to geographic region and transmission category. Therefore, dyads are more likely to be supported by subsequent partner services interviews than are genetic links found among moderate (size = 3–9) or large clusters (size ≥ 10). Similarly, gender was assortative for dyads, disassortative for clusters of size = 3–9 and ≥ 10, but assortative at both distance thresholds for all cluster sizes. This suggests that the risk and demographic profiles of small clusters are distinct from that of moderate and large clusters. For example, gender disassortativity paired with non-assortativity among transmission categories, suggesting that moderately sized clusters typically involve both genders and multiple transmission modalities. Strong assortativity by gender for larger clusters (size ≥ 10) is likely driven by the prevalence of MSM in these clusters. Notably, the transmission category is strongly non-assortative for large clusters (≥10 members), even under a stringent distance threshold (d ≤ 0.5%), suggesting that rapid-growth clusters gain members through multiple risk modalities. Combined, the assortativity results indicate initial local transmission of HIV followed by regional spread within Bulgaria via bridging to additional risk behavior groups. 

Global phylogenetic analysis was used to help explore the origin of subtype B infections in Bulgaria. Our analysis showed that there were multiple introductions of subtype B into Bulgaria from at least 8 different countries, which then spread amongst Bulgarians, including within three large clusters with 14, 67, and 552 Bulgarian members. Interestingly, HIV-1 subtype B sequences from Israel from 2005–2013 were frequently associated with Bulgarian sequences in our study [39]. Most subtype B-infected participants in that study were Israeli-born (73%), reporting high MSM exposure (83%) and suggesting possible MSM interactions between Israel and Bulgaria. The close historical relationship of citizens from both countries may reflect several events, for example the rescue of about 50,000 Bulgarian Jews to Israel during World War II [40]. In addition, Bulgaria is in the Balkans and its diverse geography, including beaches along the Black Sea, four mountain ranges, and rich cultural history, attracts visitors from Israel, neighboring countries, and Europe. These shared common origin characteristics and conditions for contemporary contacts may help to explain the observed evolutionary history of subtype B in these populations. Tourism in Bulgaria may also help to explain our finding of HIV-1 *pol* sequences from Germany, Spain, the U. K., and Russia that were ancestral to Bulgarian *pol* clades [13,14,41]. However, we cannot exclude that Bulgarians acquired their infections in these or other countries and introduced them into Bulgaria when they returned from abroad. Indeed, 78 persons with subtype B viruses reported acquiring their infections outside of Bulgaria, of which the majority (63/78, 80.8%) clustered within the large 552 taxa Bulgarian clade, two of which were ancestral to subclades with 96 and 12 members. Assuming adequate sampling and our finding of only one non-Bulgarian sequence from Cyprus in this large clade, these results do not support the hypothesis of recent importation of HIV-1 into Bulgaria as the main driver of the subtype B epidemic, unless they were all infected by an unidentified common source [42]. Nonetheless, these results may be influenced by recall bias for the potential place of infection.

Although we identified a variety of antiretroviral DRMs in the subtype B-infected population, as in our previous report but consisting of lower numbers of sequences, most DRMs were in individuals who did not cluster or were in transmission pairs [15]. Two clusters with 16 and nine members, mostly MSM, each contained NNRTI (E138G and/or V179E or K101E) or PI (L90M) DRMs, respectively, at *d* = 1.5%. However, these clusters with DRMs disassociated at d = 0.5% into singletons or pairs at *d* = 0.5%, indicating that these resistance strains are not spreading rapidly in Bulgaria, which is re-assuring. The L90M mutation is associated with a high transmission fitness, which may explain its spread as has been observed in the US and other countries [43]. 

Our study also has some other potential limitations. Although this is the most comprehensive study of all HIV-1 subtype B sequences from Bulgaria through 2018, our study population included only persons from whom the HIV-1 *pol* gene was successfully obtained. As with all molecular epidemiologic studies, the inability to sequence HIV-1 from all infected persons, including those from persons on treatment and with low viral loads, or from persons not yet diagnosed, may affect our analyses and conclusions. This is especially true for studies examining origins of transmission using only sequence information.

## 5. Conclusions

We applied a detailed phylodynamics approach to better understand the molecular epidemiology of HIV-1 subtype B infections in Bulgaria. Our findings indicate that subtype B was introduced into Bulgaria multiple times since 1988 and then infections rapidly spread among MSM and HET males who may share MSM practices. These risk behaviors continue to spread subtype B infection in Bulgaria as evidenced by the large clusters at *d* = 0.5%. Prevention strategies should continue to include increased testing and linkage to care and treatment, as well as expanded outreach to the MSM communities.

## Figures and Tables

**Table 1:** Epidemiological characteristics of subtype B-infected study participants compared to infection with other subtypes in Bulgaria.

Characteristic	Subtype B *n* (%)	Other subtypes *n* (%)	*p* value
**Total**	663	819	
**Gender**			< 0.0001
Men	593 (89.4)	612 (74.7)	
Women	70 (10.6)	207 (25.3)	
**Age (years)**			< 0.0001
≤19	23 (3.5)	68 (8.3)	
20–29	253 (38.2)	335 (40.9)	
30–39	256 (38.6)	247 (30.2)	
40–49	92 (13.9)	108 (13.2)	
≥50	39 (5.9)	61 (7.5)	
**Country of Origin**			0.007
Bulgaria	651 (98.2)	784 (95.7)	
Other country	12 (1.8)	35 (4.3)	
**Likely Country of Infection**			0.195
Bulgaria	585 (88.2)	704 (86.0)	
Other country	78 (11.8)	115 (14.0)	
**Region in Bulgaria**			< 0.0001
Sofia	368 (55.5)	311 (38.0)	
Other city	295 (44.5)	508 (62.0)	
**Transmission category^2^**			< 0.0001
HET	256 (38.6)	365 (44.6)	
MSM	376 (56.7)	166 (20.3)	
PWID	22 (3.3)	253 (30.9)	
Other	9 (1.4)	35 (4.3)	

(1) Percentages are provided in parentheses. (2) HET, heterosexual; MSM, men who have sex with men; PWID, persons who inject drugs; Other includes MSM + PWID (persons who reported both MSM and PWID); mother-to-child (MTC), and blood transfusion (BLD). (3) Fisher’s exact test was applied to Country of Origin and Transmission Category variables. Chi-squared tests were applied otherwise.

**Table 2:** Characteristics of HIV-1 subtype B clusters and unclustered persons in Bulgaria, 1988–2018, using a genetic distance cutoff of 1.5%^1^.

	Cluster Sizes	Male	Female	MSM^2^	HET	PWID	Blood Tx^3^	Vertical	Mean/Median age at Diagnosis	Likely Country of Infection (Bulgaria)	Likely Country of Infection (other)^3^	Drug Resistance Mutations (PR/NRTI/NNRTI)^4^
	91	90	1	73	16	2	0	0	34.1/32	83	8	1/0/5
	59	59	0	43	16	0	0	0	32.4/30	54	5	1/0/5
	56	52	4	31	22	3	0	0	32.7/31	51	5	0/0/3
	48	47	1	43	4	1	0	0	31.5/30	46	2	0/0/3
	16	16	1	15	1	0	0	0	33.2/33	15	1	0/0/14
	10	4	6	2	8	0	0	0	30/29.5	9	1	0/0/1
	10	9	0	4	6	0	0	0	31.7/31	10	0	0/0/3
	9	8	1	8	0	1	0	0	27.4/28	9	0	0/0/0
	9	9	0	7	2	0	0	0	30.8/30	6	3	9/0/2
	9	9	0	4	5	0	0	0	33.3/32	8	1	0/0/0
	7	7	0	7	0	0	0	0	30/32	7	0	0/0/0
	6	6	0	4	2	0	0	0	36.5/35	5	1	0/0/1
	6	6	0	6	0	0	0	0	26/26	4	2	0/1/0
	5	5	0	4	1	0	0	0	24.8/23	5	0	0/0/0
	4	4	0	3	1	0	0	0	28.3/26.5	4	0	0/0/0
	4	4	0	4	0	0	0	0	33/33	3	1	0/0/0
	4	4	0	4	0	0	0	0	44.3/44.5	3	1	0/0/0
	7 Triads (21 total)	21	0	12	9	0	0	0	30.8/27	18	3	0/0/5
	28 Dyads (56 total)	47	9	24	25	5	2	0	35.6/36	51	5	0/4/13
	Singletons (233 total)	185	48	79	138	10	2	4	32.6/32	194	39	17/36/42
**Totals**	663	593	70	377	256	22	4	4	32.7/31	585	78	28/41/97

(1) Clusters of sizes ≤3 and singletons are grouped. MSM, men who have sex with men; HET, heterosexual transmission; PWID, people who inject drugs; Vertical, mother-to-child transmission. (2) MSM includes one person reporting MSM and PWID. (3) Other countries include Australia (*n* = 1), Austria/Hungary (*n* = 1), Brazil (*n* = 1), China (*n* = 1), Cyprus (*n* = 1), Cyprus/Italy/Turkey (*n* = 1), Dominican Republic (*n* = 1), Egypt (*n* = 1), England (*n* = 2), France (*n* = 2), Germany (*n* = 13), Germany/Romania (*n* = 1), Greece (*n* = 8), Italy (*n* = 9), Italy/Spain (*n* = 1), Japan (*n* = 1), Macedonia (*n* = 4), Netherlands (*n* = 3), Republic of South Africa (*n* = 1), Scotland (*n* = 1), Spain (*n* = 13), United Kingdom (*n* = 3), United Arab Emirates (*n* = 1), Unknown (*n* = 3), USA (*n* = 3), and Zambia (*n* = 1). (4) PI, protease inhibitors; NRTI, nucleoside reverse transcriptase inhibitors; NNRTI, non-nucleoside reverse transcriptase inhibitors.

**Table 3:** Characteristics of HIV-1 subtype B clusters and unclustered persons in Bulgaria, 1988 – 2018, using a genetic distance cutoff of 0.5%^1^.

	Cluster Sizes	Male	Female	MSM	HET	PWID	Blood Tx^2^	Vertical	Mean/Median age at Diagnosis	Likely Country of Infection (Bulgaria)	Likely Country of Infection (other)^3^	Drug Resistance Mutations (PR/NRTI/NNRTI)^4^
	33	33	0	26	7	0	0	0	34.4/34	30	3	0/0/0
	22	22	0	17	5	0	0	0	32.9/31.5	19	3	0/0/1
	16	16	0	14	2	0	0	0	31.1/31.5	16	0	0/0/0
	11	10	1	8	3	0	0	0	38.8/40	11	0	0/0/0
	7	6	1	6	0	1	0	0	26/26	7	0	0/0/0
	4	4	0	4	0	0	0	0	28.8/27.5	4	0	0/0/0
	4	4	0	3	1	0	0	0	23/22.5	4	0	0/0/0
	4 Triads (12 total)	10	2	6	6	0	0	0	30.8/30.5	10	2	0/0/1
	23 Dyads (46 total)	41	5	36	10	0	0	0	34.6/32.5	40	6	2/1/10
	Singletons (508 total)	447	61	257	222	21	4	4	32.6/31	444	64	26/40/85
**Totals**	663	593	70	377	256	22	4	4	32.7/31	585	78	28/41/97

(1) Clusters of sizes ≤3 and singletons are grouped. MSM, men who have sex with men; HET, heterosexual transmission; PWID, people who inject drugs; Vertical, mother-to-child transmission; Tx, transfusion. (2) MSM includes one person reporting MSM and PWID. (3) Other countries include Australia (*n* = 1), Austria/Hungary (*n* = 1), Brazil (*n* = 1), China (*n* = 1), Cyprus (*n* = 1), Cyprus/Italy/Turkey (*n* = 1), Dominican Republic (*n* = 1), Egypt (*n* = 1), England (*n* = 2), France (*n* = 2), Germany (*n* = 13), Germany/Romania (*n* = 1), Greece (*n* = 8), Italy (*n* = 9), Italy/Spain (*n* = 1), Japan (*n* = 1), Macedonia (*n* = 4), Netherlands (*n* = 3), Republic of South Africa (*n* = 1), Scotland (*n* = 1), Spain (*n* = 13), United Kingdom (*n* = 3), United Arab Emirates (*n* = 1), Unknown (*n* = 3), USA (*n* = 3), and Zambia (*n* = 1). (4) PI, protease inhibitors; NRTI, nucleoside reverse transcriptase inhibitors; NNRTI, nonnucleoside reverse transcriptase inhibitors.

**Table 4:** Assortative mixing of subtype B clusters in Bulgaria by region, gender, age, and transmission category^1^**.**

	Cluster size, genetic distance (*d*) threshold, total persons																								
	Size	*d^2^*	Total	Size	*d*	Total	Size	*d*	Total	Size	*d*	Total	Size	*d*	Total	Size	*d*	Total	Size	*d*	Total	Size	*d*	Total	
Cluster characteristic	dyad	0.5	46	dyad	1.5	56	3–9	0.5	27	3–9	1.5	84	≥10	0.5	82	≥10	1.5	290	All	0.5	155	All	1.5	430	
Region within Bulgaria	**0.3442**			**0.6452**			−0.0316			0.0626			−0.0414			−0.0062			−0.0158			0.0038			
Gender	**0.3268**			0.0733			**−0.1111**			−0.0261			−0.0072			**0.1874**			**0.1166**			**0.1767**			
Age	0.0686			**0.2982**			0.0495			**0.2536**			0.0287			0.019			0.0606			0.0318			
Transmission category	**0.4889**			**0.6474**			−0.0934			0.0511			**−0.185**			0.0198			0.0066			0.0311			

(1) Assortativity coefficient (*r*) values of 1.0 indicate perfect assortativity, while at r = −1.0 the network is completely disassortative, and at r = 0 the network is non-assortative. Values in bold are considered significant. (2) d, genetic distance percentage.

## Supplementary Materials

Supplementary materials can be found at https://www.mdpi.com/1999-4915/12/4/441/s1.
