# Supplementary material for: Molecular Epidemiology of the HIV-1 Subtype B Sub-Epidemic in Bulgaria
Source: Viruses. 2020 Apr 14;12(4):441. doi: 10.3390/v12040441 (PMC7232140; doi:10.3390/v12040441)
Supplement: Supplementary file 1 [file viruses-12-00441-s001.zip › Supplementary files/Supplementary Table S2. 663 Bulgarian HIV-1 polymerase (pol) sequence GenBank accession numbers.docx]

**Supplementary Table S2. 663 Bulgarian HIV-1 polymerase (pol) sequence GenBank accession numbers**

**196 accession numbers for *pol* sequences from previous studies:**

1.

EF517412, EF517414, EF517417- EF517420, EF517422, EF517424, EF517426, EF517427, EF517432- EF517434, EF517436, EF517437, EF517446, EF517451- EF517453, EF517456, EF517459, EF517460, EF517462, EF517467, EF517472- EF517474, EF517478, EF517481, EF517482, EF517484, EF517485, EF517488, EF517489,

2

JQ259068, JQ259073, JQ259074, JQ259076, JQ259078, JQ259082, JQ259086, JQ259088, JQ259090, JQ259095, JQ259096, JQ259100, JQ259102, JQ259112- JQ259114, JQ259118- JQ259120, JQ259123, JQ259125, JQ259127, JQ259129- JQ259133, JQ259135, JQ259137, JQ259152- JQ259155, JQ259160- JQ259162, JQ259164, JQ259165, JQ259169, JQ259170, JQ259174, JQ259175, JQ259178- JQ259181, JQ259183, JQ259184,

3

KJ765396, KJ765397, KJ765406, KJ765408, KJ765413, KJ765417, KJ765419- KJ765421, KJ765424, KJ765428, KJ765436, KJ765441, KJ765442, KJ765446, KJ765449, KJ765451, KJ765452, KJ765456- KJ765459, KJ765464, KJ765466, KJ765467, KJ765475, KJ765477, KJ765478, KJ765479, KJ765485, KJ765491, KJ765494, KJ765496, KJ765499, KJ765503, KJ765504, KJ765507, KJ765509, KJ765511, KJ765521- KJ765524, KJ765529, KJ765531- KJ765537, KJ765539- KJ765542, KJ765544, KJ765548, KJ765553, KJ765555, KJ765558, KJ765559, KJ765562, KJ765566, KJ765567, KJ765571, KJ765575, KJ765577, KJ765584, KJ765595, KJ765599, KJ765604, KJ765605, KJ765607,

4

KT805908,

5

KY658483- KY658493, KY658495- KY658499, KY658501, KY658502, KY658504- KY658523, KY658525, KY658526,

**467 accession numbers for new *pol* sequences generated in the current study:**

MN977940, MN977944, MN977945, MN977952, MN977964, MN977966, MN977970, MN977973, MN977982, MN977988, MN977989, MN977991, MN977996, MN978006, MN978007, MN978032, MN978034, MN978039, MN978040, MN978043, MN978044, MN978046-MN978051, MN978053- MN978057, MN978060, MN978061, MN978063- MN978065, MN978068- MN978070, MN978073- MN978076, MN978078-MN978082, MN978084- MN978088, MN978090, MN978092-MN978104, MN978107- MN978114, MN978116- MN978185, MN978187-MN978204, MN978206-MN978237, MN978239- MN978281, MN978283, MN978284, MN978286- MN978293, MN978295-MN978298, MN978300- MN978371, MN978373-MN978376, MN978378-MN978449, MN978451-MN978506, MN978513, MN978521, MN978523, MN978526, MN978531, MN978567, MN978574, MN978582, MN978584, MN978594

**References**

1. Salemi, M.; Goodenow, M.M.; Montieri, S.; de Oliveira, T.; Santoro, M.M.; Beshkov, D.; Alexiev, I.; Elenkov, I.; Elenkov, I.; Yakimova, T., Varleva, T.; Rezza, G.; Ciccozzi, M. The HIV type 1 epidemic in Bulgaria involves multiple subtypes and is sustained by continuous viral inflow from West and East European countries. *Aids Res. Hum. Retrovir.* **2008**, 24:771-779.
2. Alexiev, I.; Beshkov, D.; Shankar, A.; Hanson, D.L.; Paraskevis, D.; Georgieva, V.; Karamacheva, L.; Taskov, H.; Varleva, T.; Elenkov, I. Stoicheva, M.; Nikolova, D.; Switzer W.M. Detailed Molecular Epidemiologic Characterization of HIV-1 Infection in Bulgaria Reveals Broad Diversity and Evolving Phylodynamics. *PLoS ONE* **2013**, 8.3: e59666.
3. Alexiev, I.; Shankar, A.; Wensing, A.M.J.; Beshkov, D.; Elenkov, I.; Stoycheva, M.; Nikolova, D.; Nikolova, M.; Switzer W.M. Low HIV-1 transmitted drug resistance in Bulgaria against a background of high clade diversity. *J. Antimicrob. Chemoth.* **2015**, 70.6: 1874-1880.
4. Alexiev, I.; Shankar, A.; Dimitrova, R.; Gancheva, A.; Kostadinova, A.; Teoharov, P.; Golkocheva, E.; Nikolova, M.; Muhtarova, M.; Elenkov, I.; Stoycheva, M.; Nikolova, D. Varleva, T.; Switzer W.M. Origin and spread of HIV-1 in persons who inject drugs in Bulgaria. *Infect. Genet. Evol.* **2016**, 46:269-278.
5. Alexiev, I.; Presti, A.L.; Dimitrova, R.; Foley, B.T.; Gancheva, A.; Kostadinova, A.; Nikolova, L.; Angeletti, S.; Cella, E.; Elenkov, I.; Stoycheva, M.; Nikolova, D.; Doychinova, T.; Pekova, L.; Ciccozzi. M.; Origin and Spread of HIV-1 Subtype B Among Heterosexual Individuals in Bulgaria. *Aids Res. Hum. Retrovir.* **2018**, 34.3: 244-253.
